# A pilot study of the use of human amniotic membrane as subcutaneous implants in a mouse model: a potential for temporary substitutes in two-stage breast reconstructions

**DOI:** 10.1186/s12905-023-02531-9

**Published:** 2023-07-12

**Authors:** Sadaf Alipour, Ramesh Omranipour, Bita Eslami, Solmaz Khalighfard, Azin Saberi, Azar Shabestari, Ali Mohammad Alizadeh

**Affiliations:** 1 Breast Disease Research Center, Cancer Institute, Tehran University of Medical Sciences, Tehran, Iran; 2 Department of Surgery, Arash Women’s Hospital, Tehran University of Medical Sciences, Tehran, Iran; 3 Department of Surgical Oncology, Cancer Institute, Tehran University of Medical Sciences, Tehran, Iran; 4 Research Center on Developing Advanced Technologies, Tehran, Iran; 5 Nursing Division, Arash Women’s Hospital, Tehran University of Medical Sciences, Tehran, Iran; 6 Cancer Research Center, Cancer Institute, Tehran University of Medical Sciences, Tehran, Iran

**Keywords:** Amniotic membrane, Biological dressing, Biomaterial, Breast implant, Reconstruction, Mice

## Abstract

**Introduction:**

Breast reconstruction by prosthesis is frequently performed in breast cancer treatments, and a temporary substitute is used in the first step of two-stage operations.

**Aim:**

Due to the advantageous biological features of the human amniotic membrane, we aimed to evaluate its use for temporary implants.

**Method:**

We prepared small spherical implants from human amniotic membranes and inserted them into BALB/c mice’s subcutaneous flanks. Then, we compared the bulging they produced, the durability, and the host reaction with implants made from the chorionic membrane, folded membrane patches, and sterile plastic beads.

**Results:**

All amionitic cases were healthy throughout the study and only mild inflammation occurred in them. Furthermore, the bulging of the implants was acceptable and faded gradually. However, moderate inflammation was observed in chorionic implant mice, and the bulging disappeared very soon. Finally, the control group had severe inflammation and the beads implant was rejected.

**Conclusion:**

Our study showed that the human amniotic membrane could represent a safe and valid tool for breast reconstruction, however, further studies on larger animals and more implants are suggested.

## Introduction

Breast cancer is the most common female cancer worldwide [1]. Breast reconstruction is one of the procedures performed very frequently in the treatment of this malignancy in women who undergo a mastectomy. Tissue flaps can be used for the reconstruction of different body parts, but this is not possible in all patients because of insufficient tissue or bulk. Therefore, reconstruction is most commonly accomplished using breast prostheses. This might be performed as a two-stage procedure [2]; in the first stage, the breast skin is preserved while a skin-sparing mastectomy or nipple-sparing mastectomy is performed. A tissue expander or temporary implant is inserted below the skin flaps. The second stage is performed later by exchanging the tissue expander for a permanent breast prosthesis. All implants are made of synthetic materials, which may be rejected by the body’s defense mechanisms. Biomaterials are now recognized as more compatible with living organisms than synthetic materials and find extensive use in repair or reconstructive procedures. Amniotic membranes are widely available at low cost, have no blood or lymphatic vessels or nerves, and are fed by diffusion of nutrients; these interesting biologic features make them an attractive choice for body organ substitutes [3]. Thus, we studied whether human amniotic membranes could be used as temporary implants in the first stage of breast reconstruction or not. In this regard, we had several objectives, and we designed the cases and controls according to them: (1) to assess the potential of the amniotic membrane for inducing inflammatory reactions in comparison with control materials regardless of the implant shape, (2) to check the feasibility of shaping the amniotic membrane for breast implants, (3) to evaluate the volume or amount of bulging provided by the implants, and (4) to follow their durability.

## Materials and methods

### Settings

The objectives were set in the plan. For the first objective, we used the chorionic membrane as a biological control and sterile plastic beads as synthetic controls. Targeting the second goal, we created spherical implants from the amniotic membrane, and from the chorionic layer as controls. To answer our third query about the prominence produced by the implants, we prepared unshaped folded patches of the membranes to insert into the controls. The plastic beads also helped to ascertain if the bulging could be caused by an unrelated foreign material. The last aim was to compare amniotic implants and controls regarding bulging duration.

### Membrane extraction and processing

Research goals were explained to women undergoing an elective cesarean section of a term baby. All women had been tested and were negative for the Human Immunodeficiency Virus and Hepatitis B and C viruses, and none had any previous history of underlying disease; the fetus screening tests were also normal.

The placenta and the amniotic sac were extracted as intact as possible by the obstetricians while performing the cesarean surgery in a sterile container, and the membranes were removed.

**Fig. 1:**
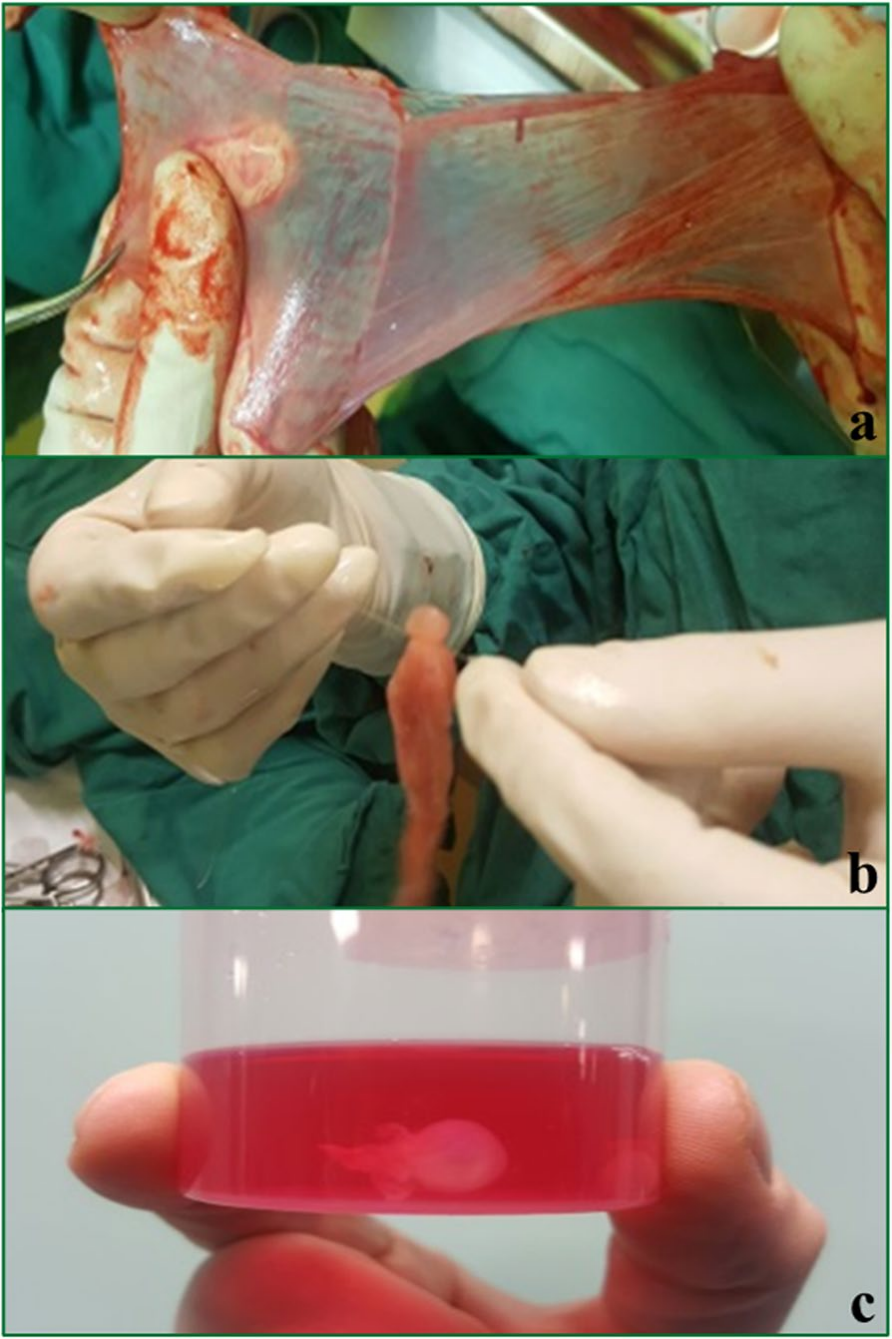
**a** Isolation of the amniotic and chorionic layers, **b** ligation of the proximal part of a piece of material after being inflated by injection, and **c** implant spheres soaked in phosphate-buffered saline

The amniotic and chorionic layers of the fresh membranes were separated (Fig. 1a). First, the membranes were extensively washed with sterile normal saline to remove blood clots and debris. They were then washed in a phosphate-buffered saline (PBS) solution containing 100 U/ml penicillin G, 100 µg/mL streptomycin, and 0.25 µg/mL amphotericin B. They were then soaked in PBS and waited to be transformed into implants.

### Implant preparation

Both the amniotic and chorionic layers of the membranes were cut into different sizes. Several small folded patches were prepared. One-layer chorionic or amniotic membranes were used for implants. They were placed on the tip of a syringe containing PBS or normal saline, and a small amount of the solution was injected. The proximal part of the membrane was ligated by a 4 − 0 monocrystal suture while it was inflated by injection, and the tails were trimmed (Fig. 1b). All procedures were performed under sterile conditions. The implant spheres and the membrane patches were soaked in PBS (Fig. 1c) and immediately transported to the animal laboratory in an icebox.

### Animal grouping and procedures

Ten female BALB/c mice (aged 8–10 weeks, purchased from the Pasteur Institute of Iran) were divided into four groups: (1) Case group, including 3 mice who received a subcutaneous spherical amniotic implant; (2) Control group A, including 2 mice without any treatment; (3) Control group B, including 3 mice with biological implants (amniotic folded patch, chorionic spherical implants or folded patches); (4) Control group C, including 2 mice with plastic beads.

Anesthesia was first administered by intraperitoneal injection of Xylazine (10 mg/kg) and Ketamine (100 mg/kg). The surgery was then performed supine via 2–5 mm incisions in shaved skin. To create a space for the sphere implants, a small subcutaneous pocket was created lateral to the incision by blunt dissection with a small clamp. This is to ensure that the incision is not placed just above the cavity. Smaller pockets were created for membrane patches. The folded patches or spherical implants were pushed inside the subcutaneous pockets through the incisions. These pockets were closed by 1 to 3 separate sutures.

### Measurements

The mice were weighed on days 1, 8, 15, and 22 after surgery. General conditions and deaths were recorded. Implant volume was weekly measured on days 1, 8, 15, 22, and 29 until the bulge flattened by a Digital Vernier Caliper (Mitutoyo, Japan) based on the formula: V = 1/6 (π L W D); where V = volume, L = Length, W = Width, and D = Depth. Local inflammation at the implant site was rated by the team’s animal experts according to the extent of redness and swelling.

## Results

The type of implant in each mouse, local reactions to the implants, and weight of mice are described in detail in Table 1. In the case group, the mice were healthy throughout the follow-up time. Mild inflammation occurred on the first day in all of them but resolved completely within one week. The implant bulge was prominent and acceptable in this group. It faded gradually after around two weeks except in mice with a folded amniotic implant. Their bulge flattened quickly, probably due to tissue necrosis. The mice with chorionic implants were mildly ill. There was a moderate inflammatory reaction at the surgical site in both the chorionic patch and chorionic spheres. A noticeable bulging was observed, but it quickly faded away. As soon as the non-healing incisions healed, the plastic beads were rejected and removed. The two mice in this group were moderately ill and lost weight in the first week. They were immediately euthanized and exited from the study via cervical dislocation.

**Table 1:**
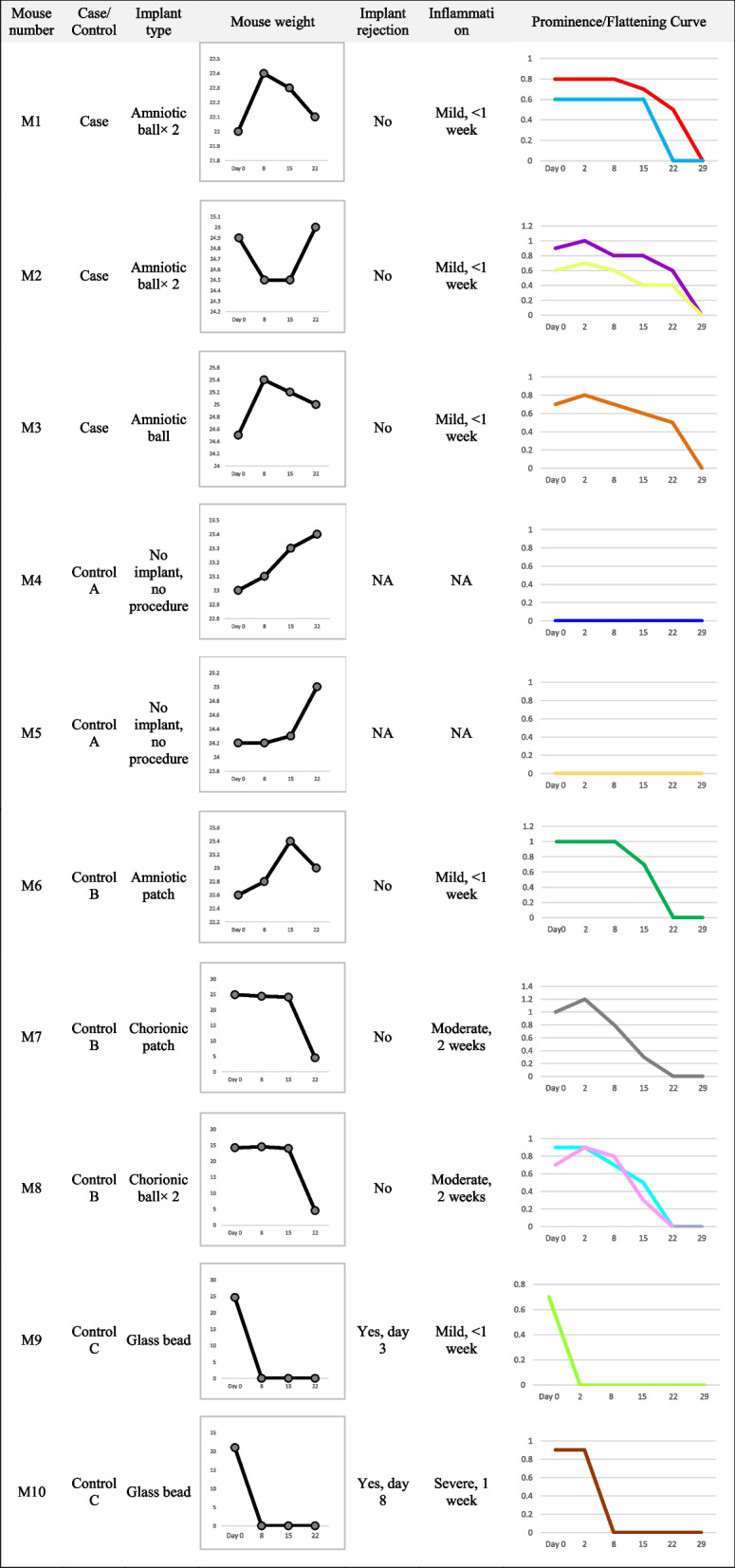
Characteristics of mice and implants, course of changes in mice weight and size of bulging produced by implants in cases and controls

## Discussion

We assessed the possibility of using the amniotic membrane as a temporary subcutaneous implant in mice. Our results show that this can be seen as an acceptable option for this purpose. It seems that this is the first study to apply the human amniotic membrane as a biomaterial for prosthesis purposes. So far, the amniotic membrane has been studied for different medical purposes. It has been used as a biological dressing in chronic wounds, particularly diabetic foot ulcers [4], for effective coverage of skin burns [5], temporary coverage in corneal abrasions or ulcers and bullous keratopathy [6], and for repairing tissue defects such as scalp defects [7] and nerve damages [8]. Providing bulk by taking advantage of the amniotic membrane is a field not yet studied. Cell culture or even folding large amounts of the amniotic membrane might produce the desired volume. In our study, the bulge provided by the amniotic membrane spheres was satisfactory. The comparison with the bulk and course of prominence occurring in controls shows the probable suitability of the amniotic membrane for achieving this goal. However, the question of vascularization and feeding of the produced bulk, its probable weight, and the need for advanced technology can prohibit the extensive use of such implants. Hence, we used the membrane as a container and assessed whether it was suitable for this purpose.

No disturbing reactions or adverse effects occurred at the surgical sites or in the implant recipients. The comparison of the local inflammation and overall health of the mice in the case group with the controls shows that the amniotic membrane is a compatible material. It can probably be used for prosthetic purposes. In addition, no rejection events occurred. These results were close to our expectations and suggested a favorable outlook for amniotic membrane breast implants. However, some critical issues need to be explored to establish the potential role of the amniotic membrane in this proposed setting: the tolerance of the membrane for high amounts of fluid, the best media used for filling a larger implant, and the durability of the prominence produced by it, and the strength of the implant in cases of stress or trauma. These must be investigated with larger amniotic implants in larger laboratory animals.

Our study also had some limitations. Considering the human nature of the amniotic membrane, mice were not immunosuppressed. We did not examine the histologic changes in the subcutaneous cavities after implant absorption or rejection and the alterations in the amniotic membrane at the cellular level. Likewise, the very small size of the balls interfered with sound judgments about volume and durability. Further studies on larger laboratory animals with larger implants are warranted.

## Conclusion

Our results showed that the human amniotic membrane could represent a safe and valid tool for breast reconstruction, however, the processing, method of preparation, and ways of shaping these prostheses need to be explored. A further animal study on larger animals, as well as a technology investigation, is recommended. We are doing this to confirm safety and explore technical considerations.

## Data Availability

The data that support the findings of this study are available on request from the corresponding author [Ali Mohammad Alizadeh].
